# Long-Lifetime Ag/AgCl Electrodes Prepared by Pulse Current Electrodeposition for Chloride Monitoring in the Concrete Environment

**DOI:** 10.3390/s25165032

**Published:** 2025-08-13

**Authors:** Xiangyu Lu, Jing Hu, Xingguo Feng, Qiyan Zhou, Zhanqing Qu, Jisheng Zhang, Ruihu Zhu, Huaqing Zhang, Songgui Chen

**Affiliations:** 1 Key Laboratory of Coastal Disaster and Defence, Ministry of Education, Hohai University, Nanjing 210024, China; luxiangyu@hhu.edu.cn (X.L.); hj2623726871@163.com (J.H.); 18261933165@163.com (Q.Z.); qu18916267627@163.com (Z.Q.); jszhang@hhu.edu.cn (J.Z.); 20090061@hhu.edu.cn (R.Z.); 2 Tianjin Research Institute for Water Transport Engineering, Ministry of Transport, Tianjing 300456, China; tjzhq1@163.com (H.Z.); chensg@tiwte.ac.cn (S.C.)

**Keywords:** Ag/AgCl electrode, pulse current electrodeposition, constant current electrodeposition, chloride ion monitoring

## Abstract

Lifetimes of Ag/AgCl electrodes determine whether it is possible to monitor the concentration of chloride ions in marine concrete structures. A novel manufacturing method, pulse current electrodeposition at a low current density, was proposed to prepare the long-lifetime Ag/AgCl electrode. Influences of electrodeposition duration were investigated on the Nernst response, exchange current density, and lifetime of Ag/AgCl electrodes, and the properties were also compared to those of the ones electrodeposited by applying constant currents. Ag/AgCl electrodes prepared with the pulse current exhibited a wider potential response, a higher exchange current density, and a longer lifetime than those prepared by the constant current under the same equivalent charge transfer conditions. AgCl film on the electrode prepared with the pulse current displayed a thicker layer, a lower density of micropores, a higher Cl/O ratio, and a lower Ag/Cl ratio than those of its counterpart electrodeposited by applying the constant current. The lifetime of the Ag/AgCl electrode was mainly determined by the thickness of AgCl films in the concrete environment. The lifetimes of the Ag/AgCl electrode, which was prepared with a 0.1 mA cm^−2^ pulse current for 15 h, were 420 h in pore solution and more than 3500 h in mortar, respectively. In addition, the potential of this Ag/AgCl electrode did not show any significant decrease after 3500 h in the mortar without Cl^−^. The results suggest that pulse current electrodeposition is an effective method to improve the lifetimes of Ag/AgCl electrodes in concrete.

## 1. Introduction

Chloride ions are one of the main factors that reduce the durability of marine concrete structures [1,2]. When the concentration of chloride ions reached the critical chloride concentration at the rebar/concrete interface, the rebar started to corrode, which reduced the structural load-bearing capacity and caused cracks in the concrete protective layer for the expansion of the corrosion products [3]. These phenomena accelerated the degradation and failure of marine concrete structures [3]. Therefore, it is crucial to accurately monitor the concentration of chloride ions in the concrete protective layer, and the information can be beneficial for timely repair treatments and enhanced structural durability [4,5].

In recent years, the in situ monitoring technologies for chloride ion concentration in concrete have attracted a great deal of attention. Two types of technologies, fiber optic sensors and Ag/AgCl selective electrodes, were generally introduced in previous studies [6,7,8,9]. Fiber optic sensors need complex and expensive equipment for monitoring chloride ion concentration in concrete [6]. In comparison, the Ag/AgCl selective electrode, which is easy to produce and of high precision, was widely considered the most promising technology for monitoring chloride ions in marine concrete structures [7,8,9].

The electrode potential (φ) of Ag/AgCl selective electrodes varies with the concentration of chloride ions in environments, and its response complies with the Nernst equation under thermodynamic equilibrium, as Equation (1) shows [10].

(1) $$\varphi =\varphi_{{Ag}^{+}/\mathrm{Ag}}^{\theta }+\frac{RT}{F}lnK_{sp}-\frac{RT}{F}lnC_{{Cl}^{-}}=\varphi_{Ag/AgCl}^{\theta }-\frac{RT}{F}lnC_{{Cl}^{-}}$$

in which *φ*^θ^_Ag+/Ag_ is the standard potential of Ag electrode; *K_sp_* is the solubility product of AgCl; *R* is the natural gas constant; *T* is the temperature of the solution; *F* is the Faraday’s constant; *φ*^θ^_Ag/AgCl_ is the standard potential of Ag/AgCl electrode; and *C*_Cl_^−^ is Cl^−^ concentration on the surface of Ag/AgCl electrode.

Previous studies suggested that the properties of the Ag/AgCl electrode used for chloride monitoring were significantly influenced by its preparation methods. In general, the Ag/AgCl electrodes were prepared by applying constant current electrodeposition [11,12,13,14], dip coating [10,15,16], and sintering [4,17]. Among them, the electrodeposition method can rapidly deposit a layer of AgCl films on the Ag substrate by applying constant currents or potentials. The thickness of AgCl films significantly affected the potential response and service life of the Ag/AgCl electrode in concrete environments [8,14,18,19,20]. Earlier studies reported that a thicker AgCl film led to a longer time for the Ag/AgCl electrode to reach its stable potential [18,19]. However, the thinner AgCl film shortened the lifetime of the Ag/AgCl electrode in concrete, and this scenario was attributed to the transformation or degradation of AgCl films in the high concentration of OH^−^ in the environment [12,18]. In addition, the microstructures of the AgCl films are closely related to the performance of the Ag/AgCl electrode in concrete. In particular, the Ag/AgCl electrode with a porous AgCl film displayed high exchange current density, which means a short response time at electrochemical reaction equilibrium [21,22]. Simultaneously, the porous AgCl film facilitated the penetration of OH^−^ ions and accelerated the degradation of AgCl films, which led to the potential of the Ag/AgCl electrode’s negative shift and short lifetime [21,23]. Therefore, it is crucial to adjust the thickness and porosity of the AgCl film on the electrode to let the Ag/AgCl electrode be suitable for the in situ and long-term monitoring of chloride ions in concrete.

Previous studies have confirmed that the pulse electrochemical deposition, at a high current density (~10 mA.cm^2^), can refine the particle size and increase the density of the deposited layer, in comparison to the film electrodeposited by applying the constant current [24,25,26]. This situation was attributed to the easier nucleation under the pulse current, as well as to the grain growth being inhibited by the relaxation time in the pulse current [24,25,26]. Cao [27] and Du [28] investigated the performance of the Ag/AgCl electrode electrodeposited by applying constant current with different low current densities (~1.0 mA.cm^2^), and the better AgCl film/Ag substrate bonding interface was observed on the Ag/AgCl electrode electrodeposited by the lower current density. Simultaneously, the Ag/AgCl electrode electrodeposited at the lower current density was more sensitive to the concentration of Cl^−^ [27,28]. Aiming to develop a long-lifetime Ag/AgCl electrode in a concrete environment, electrodeposition on the Ag/AgCl electrode was attempted in the present study by applying the pulse current at low current density. Ag/AgCl electrodes were electrodeposited by applying constant current and pulse current at a low current density (0.1 mA.cm^2^, about 1/00 of previous studies [24,25,26]), respectively. The Nernst response and exchange current density of the Ag/AgCl electrodes were investigated in a simulated pore solution, and the lifetimes were separately tested in pore solution and mortar. Morphology, thickness, and the chemical composition of the AgCl films on the electrodes were measured, and the degradation mechanism of the Ag/AgCl electrodes, which was prepared by applying the pulse current electrodeposition at a low current density, also was analyzed. The results confirmed that the pulse current electrodeposition was an effective method to extend the lifetime of the Ag/AgCl electrode in concrete environments.

## 2. Experimental Methods

### 2.1. Preparation of Ag/AgCl Electrode

Pure Ag foil (purity ≥ 99.9%, Zhongyan Metal Materials Co., Ltd., Xingtai, Hebei, China), with a size of 10 mm × 10 mm × 0.5 mm, was used to prepare the Ag/AgCl electrode. A copper wire was welded on each Ag foil sample, and the welded joints were sealed with epoxy resin. Prior to the electrodeposition, the Ag foil was sequentially polished with sandpaper of grits 400#, 1000#, and 1200#. Then, the Ag foil sample was cleaned by applying ultrasonic waves in acetone for 15 min, followed by soaking in a 28% NH_4_OH (Sinopharm Chemical Reagent Co. Ltd., Shanghai, China) solution for 4 h. Afterward, it was rinsed with deionized water and immersed in concentrated nitric acid (68%, wt.%, Sinopharm Chemical Reagent Co. Ltd., Shanghai, China) for 1 min to activate the surface, followed by rinsing with a large volume of deionized water. During the electrodeposition, a Ag foil sample, a saturated calomel electrode (SCE), and a circular graphite cylinder were used as the working electrode, the reference electrode, and the counter electrode, respectively. The electrodeposition was conducted in a 0.1 M HCl solution.

Previous studies [19,29] have reported that the AgCl film displayed a good bond behavior on the Ag substrate when the electrodeposition was conducted at a lower constant current density, and the prepared Ag/AgCl electrodes exhibited a satisfactory performance. For comparison, the Ag/AgCl electrodes were electrodeposited by applying constant current and pulse current, respectively. All the electrodepositions were performed at a current density of 0.1 mA/cm^2^ [19] in the present study.

**Figure 1:**
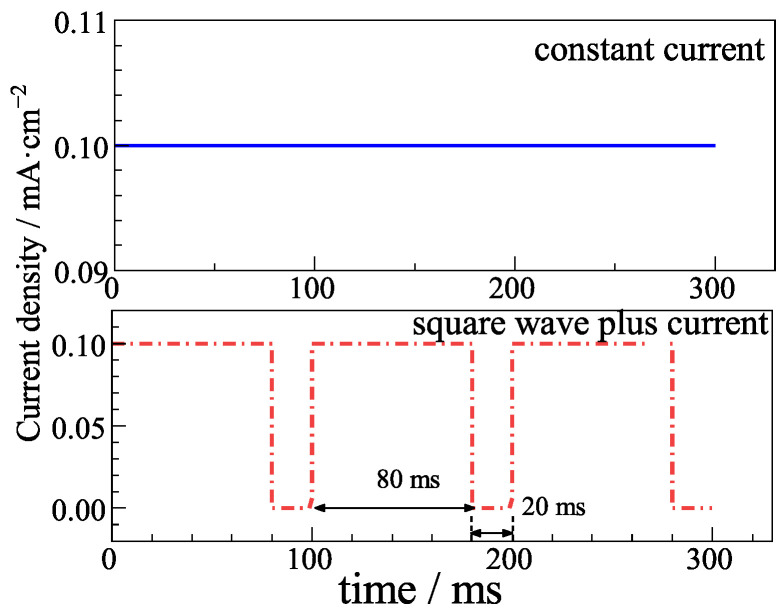
Schematic diagram of current variation over time.

A CS350 (Corrtest, Wuahan, China) electrochemical workstation was used to conduct electrodeposition and electrochemical measurements. Figure 1 shows the constant current and square wave pulse current adopted here. The duty ratio of the square wave pulse current was 80%. In each cycle of the square wave pulse current, the current density maintained at 0.1 mA/cm^−2^ for 80 ms and at 0 mA/cm^−2^ for 20 ms. Table 1 shows the current parameters applied for the Ag/AgCl electrode in electrodeposition. To ensure that the same equivalent charge transfers for the constant current and pulse current electrodeposition, as Table 1 shows, the duration for the constant current was 6 h and 12 h, while the corresponding duration for the pulse current was 7.5 h and 15 h, respectively.

### 2.2. Electrochemical Measurements

To avoid the influence of the deposition of Ca^2+^ and SO_4_^2−^ on the Nernst response of the Ag/AgCl electrode, an alkaline solution (pH value was 13.5) without Ca^2+^ and SO_4_^2−^ was adopted to simulate concrete pore solution, which consisted of 8.0 g/L NaOH (Sinopharm Chemical Reagent Co. Ltd., Shanghai, China) and 35.6 g/L KOH (Sinopharm Chemical Reagent Co. Ltd., Shanghai, China) [14]. Different concentrations of Cl^−^, including 0.001 M, 0.01 M, 0.1 M, and 1.0 M, were added to the simulated concrete pore solution. The open circuit potential (OCP) of the Ag/AgCl electrodes was measured by using the CS 350 electrochemical station in different Cl^−^ concentration solutions. When the Ag/AgCl electrodes were immersed into pore solution with different Cl^−^ concentrations to investigate its Nernst response, three electrodes were used for each condition and the average value of the OCPs was adopted in the present study. The Ag/AgCl electrodes were immersed in the simulated concrete pore solution at room temperature (20 ± 1 °C), and the OCP of the electrode was recorded every 48 h in the pore solution. The simulated concrete pore solution was refreshed every 48 h after the electrochemical measurement.

**Figure 2:**
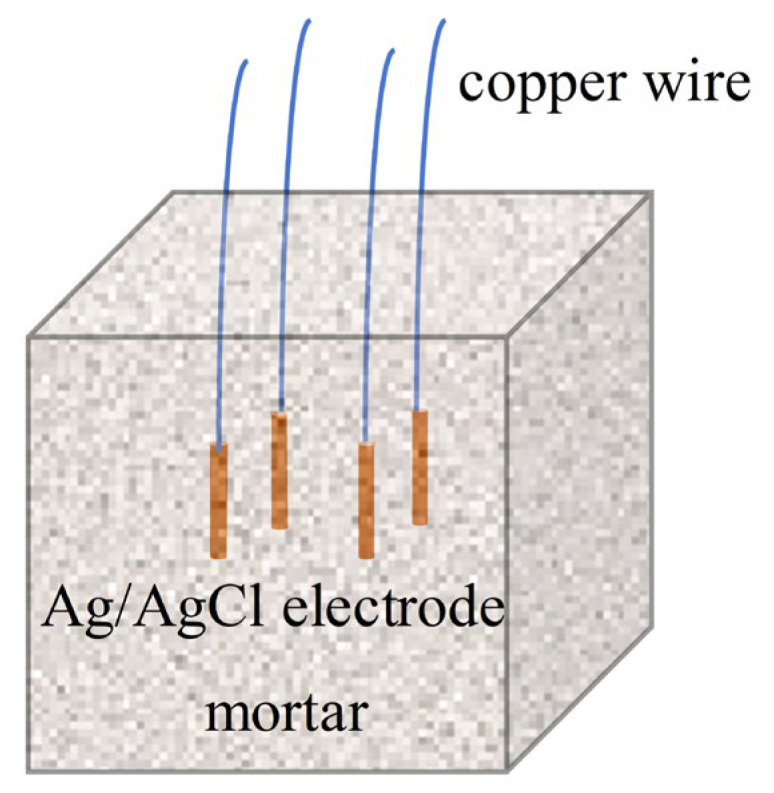
Schematic drawing of Ag/AgCl electrodes in mortar.

To investigate the performance of the Ag/AgCl electrode in a real concrete environment, Ag/AgCl electrodes were embedded in a mortar sample. Portland cement (P.O 42.5) and river sand with a fineness module of 2.6 were adopted to prepare the mortar. The cement–sand ratio of the mortar was 1:2.5 and its water–cement ratio was 0.50. The size of the mortar sample was 150 mm × 150 mm × 100 mm. As Figure 2 shows, four Ag/AgCl electrodes, prepared with different types of currents and electrodeposition time, were embedded together in the mortar. The mortar, which contained these Ag/AgCl electrodes, was removed from its mold after 24 h of casting and was cured at 95% relative humidity and 20 ± 1 °C, in a standard curing box for 28 d. Then, the Ag/AgCl electrode/mortar samples were immersed in deionized water and the OCP of each Ag/AgCl electrode was tested. It is worth mentioning that the failure of Ag/AgCl electrodes mainly came from the degradation of AgCl film in the high concentration of OH^−^ according to previous studies [12,18]. Thus, the lifetime of the Ag/AgCl electrode in the mortar without Cl^−^ was more in line with that of the electrode in the actual concrete environment.

All electrochemical measurements were conducted by applying a CS350 workstation with a typical three-electrode arrangement at room temperature (20 ± 1 °C). A saturated calomel electrode (SCE) and a platinum electrode were used as the reference electrode and the counter electrode, respectively. The OCP was considered stable if the potential fluctuation was less than 5 mV within 10 min. The potentiodynamic polarization tests were carried out from −80 to 80 mV vs. OCP at a rate of 0.5 mV/s.

### 2.3. Microstructure and Elemental Composition Analysis

A Hitachi S4800 scanning electron microscope (SEM) was utilized to observe the morphology and the cross profile of the AgCl film on the Ag/AgCl electrode. The elemental composition and distribution of the AgCl films were analyzed by using electron probe microanalysis (Oxford, X-max 80). The AgCl film was also analyzed by applying X-ray diffraction (XRD, X’Pert PRO MPD) with Cu K_α_ at 40 kV between 3° and 80°, at a scanning rate of 10°/min.

## 3. Results and Discussion

### 3.1. Sensitivity to Chloride Ion Concentration

**Figure 3:**
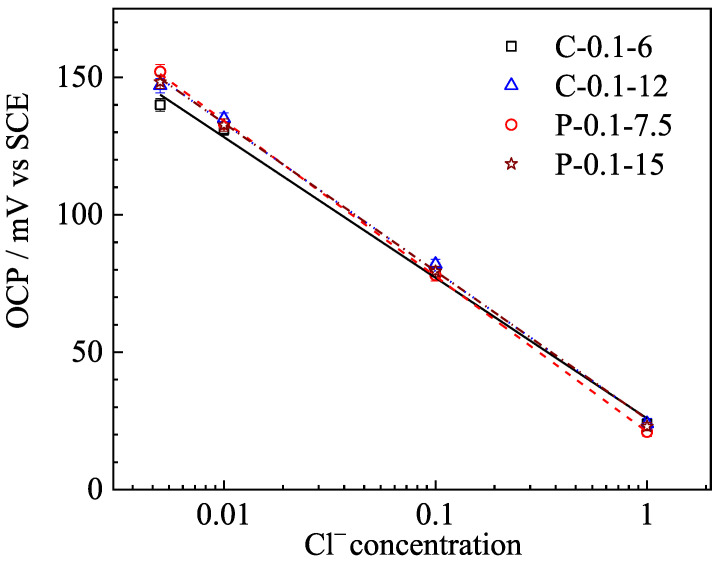
OCP response of different Ag/AgCl electrodes in the simulated pore solutions with various concentrations of Cl^−^.

Figure 3 illustrates the potentials (open circuit potential, OCP) of the Ag/AgCl electrodes in the simulated pore solutions with different concentrations of Cl^−^. It is easy to notice that the OCPs of the Ag/AgCl electrodes significantly decrease with increasing Cl^−^ concentration. In general, the OCPs of the Ag/AgCl electrodes, which were electrodeposited by applying the constant current and the pulse current, show good linear relationships with the logarithm of Cl^−^ concentrations. This situation is consistent with an earlier study [19], indicating that Ag/AgCl electrodes prepared at a small current density exhibited satisfactory Nernst response characteristics. Furthermore, with the same equivalent charge transfer in electrodeposition, the OCP change amplitude of Ag/AgCl electrodes prepared by pulse current is slightly wider (~10 mV) than those of its counterparts prepared by constant current in pore solutions with the same range of Cl^−^ concentrations.

To quantitatively analyze the Nernst response characteristics of these Ag/AgCl electrodes, linear fitting was performed between the OCP of the Ag/AgCl electrode and the logarithm of Cl^−^ concentration, and the results are listed in Table 2. Satisfactory linear relationships were observed, as evident by the high values (>0.99) of the correlation coefficients (R^2^). Furthermore, the Ag/AgCl electrode prepared with the pulse current displayed a higher value of slope than that of the electrode prepared with the constant currents. For instance, the slope of the P-0.1-7.5 electrode is −56.540, while that of the C-0.1-6 is only −51.131. This scenario suggests that the Ag/AgCl electrode prepared by the former type of current was more sensitive to the change in Cl^−^ concentration, which is beneficial for the monitoring of Cl^−^ concentration in concrete environments. As Table 2 shows, the absolute value of the P-0.1-7.5 slope was slightly higher than that of the P-0.1-15, which suggests that the sensitivity of the Ag/AgCl electrode decreased with the increasing electrodeposition time. This situation can be attributed to the thicker AgCl film of the latter sample, which results in a longer time for the Ag/AgCl electrode to achieve its stable potential, according to earlier studies [18,19].

### 3.2. Polarization Curves of Ag/AgCl Electrodes

**Figure 4:**
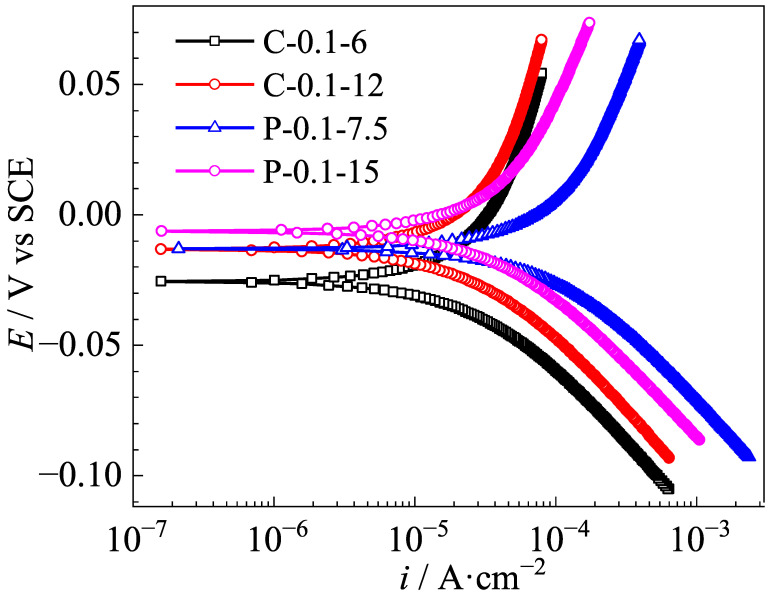
Polarization curves of the Ag/AgCl electrodes electrodeposited by different currents in the simulated pore solution with 0.05 M Cl^−^.

Polarization curves of these Ag/AgCl electrodes were tested in a simulated pore solution containing 0.05 M Cl^−^, and the exchange current densities were calculated from the Tafel slope of the polarization curves, in the range from −50 to 50 V vs. OCP. The results are displayed in Figure 4 and Table 3, respectively. According to an earlier study [29], the polarization curve reflects the kinetic characteristics of electron transfer at the electrode surface. A larger exchange current density suggests a faster rate of electron exchange between Ag and Ag^+^, which also means that the Ag/AgCl electrode has better reversibility and stronger polarization resistance. As Figure 4 shows, the corrosion potential (*E_corr_*) of the Ag/AgCl electrode prepared with the pulse current was slightly higher than that of the counterpart prepared with the constant current with the same equivalent charge transfers. Simultaneously, the exchange current density of the former Ag/AgCl electrode was obviously higher than that of the latter one (Table 3), which could be related to the thickness of the AgCl film, as well as to its microstructure. This situation indicates that the Ag/AgCl electrodes prepared by applying the pulse current have better reversibility and stronger polarization resistance than those counterparts prepared with the constant current electrodeposition [29].

### 3.3. Stability of Ag/AgCl Electrodes

**Figure 5:**
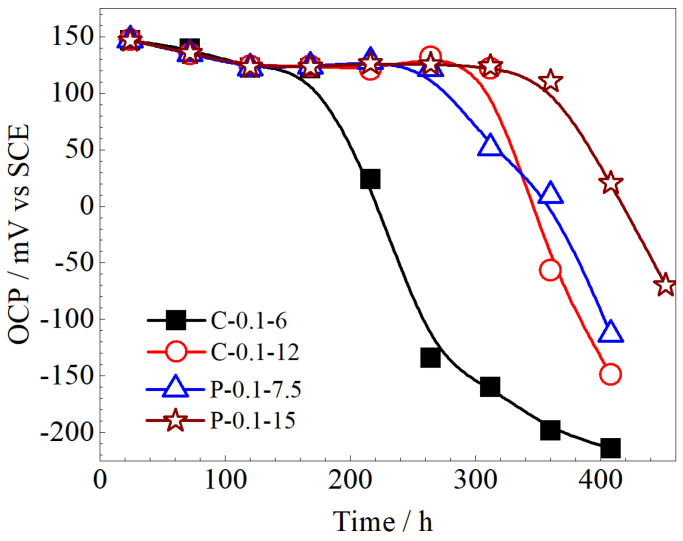
The OCP of the Ag/AgCl electrodes electrodeposited by different currents in the simulated concrete pore solutions.

With the aim of investigating the long-term stability of Ag/AgCl electrodes, the OCPs of these electrodes were recorded in chloride-free pore solution and mortar, respectively. Figure 5 presents the OCP of the Ag/AgCl electrodes electrodeposited by different currents in the simulated concrete pore solutions. It can be observed that the OCPs of all Ag/AgCl electrodes in the simulated concrete pore solution exhibit a significant decrease with immersion time. Zhang et al. [12] also noticed a sharp decrease in the OCPs of Ag/AgCl electrodes in a simulated concrete pore solution, and a decrease in OCP of more than 140 mV was defined as the failure of the Ag/AgCl electrode for the significant increase in the AgCl film polarization resistance. They attributed this phenomenon to the OH^−^ penetrating through the AgCl film and adsorption on the Ag substrate in the simulated concrete pore solution. Additionally, the gel-like substances formed by the AgCl film and pore solution generate tensile stress on the film layer, which exceeds the adhesion strength between the AgCl film and the Ag substrate, leading to both degradation and delamination of the AgCl film [12]. Thus, as Figure 5 shows, a sharp decrease can be observed in the OCPs of the Ag/AgCl electrodes. It is worth mentioning that Angst et al. [30] ascribed this sharp decrease in OCP to the transformation of AgCl to Ag_2_O in the film of Ag/AgCl electrodes in the highly alkaline concrete environment.

As shown in Figure 5, the Ag/AgCl electrodes prepared with the constant current (C-0.1-6 and C-0.1-12) exhibited a sharp decrease in OCPs after being immersed in simulated concrete pore solution for 216 h and 360 h, respectively. In comparison, the Ag/AgCl electrodes prepared by applying the pulse current (P-0.1-7.5 and P-0.1-15) showed a noticeable drop after 312 h and 408 h. This situation suggests that the stabilities of Ag/AgCl electrodes prepared by the pulse current are better than those of their counterparts prepared by the constant current under the same equivalent charge transfer conditions. Moreover, the stability duration of the Ag/AgCl electrodes increased with the time of deposition. In general, the Ag/AgCl electrode prepared by applying the pulse current deposition for 15 h (P-0.1-15) displayed the longest stable duration in the simulated concrete pore solution.

An earlier study [12] reported that the stability duration of the Ag/AgCl electrode in pore solutions was only about 1/150 of the stability time of its counterpart in cement paste. The authors [12] attributed this scenario to the simultaneous degradation and delamination of the AgCl film in the pore solution, whereas only the degradation of the AgCl film occurs in the cement paste. Thus, the lifetime of the Ag/AgCl electrode in cement paste was significantly longer than that in the simulated concrete pore solution. In the present study, the Ag/AgCl electrodes had a lifetime of more than 200 h in pore solutions, especially that of the P-0.1-15 Ag/AgCl, which reached 408 h, implying the long-term stability (3~7 years) of the Ag/AgCl electrodes in mortar according to earlier study [12].

**Figure 6:**
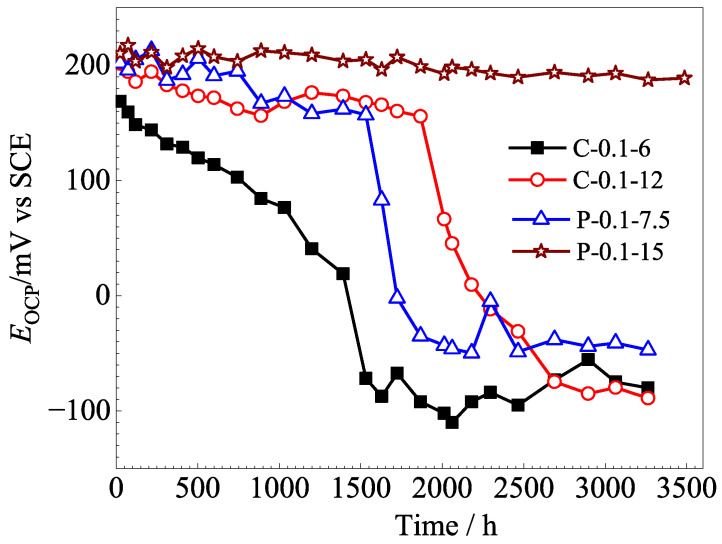
The OCP of the Ag/AgCl electrodes electrodeposited by different currents in the mortar.

The OCPs of the Ag/AgCl electrodes also were investigated in mortar, and the results are displayed in Figure 6. For the electrodes prepared by applying the constant current, the OCP of the C-0.1-6 Ag/AgCl electrode decreased by 140 mV after 1200 h in the mortar, which means that the electrode failed in 1200 h according to Zhang [12]. As the electrodeposition time increased to 12 h (C-0.1-12), the lifetime of the electrode increased to about 2000 h in the mortar. In comparison, the lifetime of the P-0.1-7.5 and P-0.1-15 electrodes, which electrodeposited by applying the pulse current with the same equivalent charge transfers, were significantly longer than those of the former electrodes, exceeding 1700 h and 3500 h, respectively. Overall, the P-0.1-15 electrode, which was prepared by applying pulse current for 15 h, exhibits the best long-term stability performance. The P-0.1-15 electrode did not show a significant decrease in OCP after 3500 h in mortar. These results suggest that the pulse current electrodeposition is an effective method for extending the lifetime of Ag/AgCl electrodes in concrete.

### 3.4. Microstructure Analysis of Ag/AgCl Electrodes

**Figure 7:**
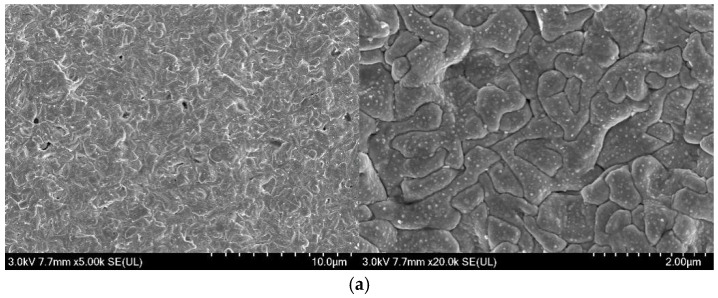

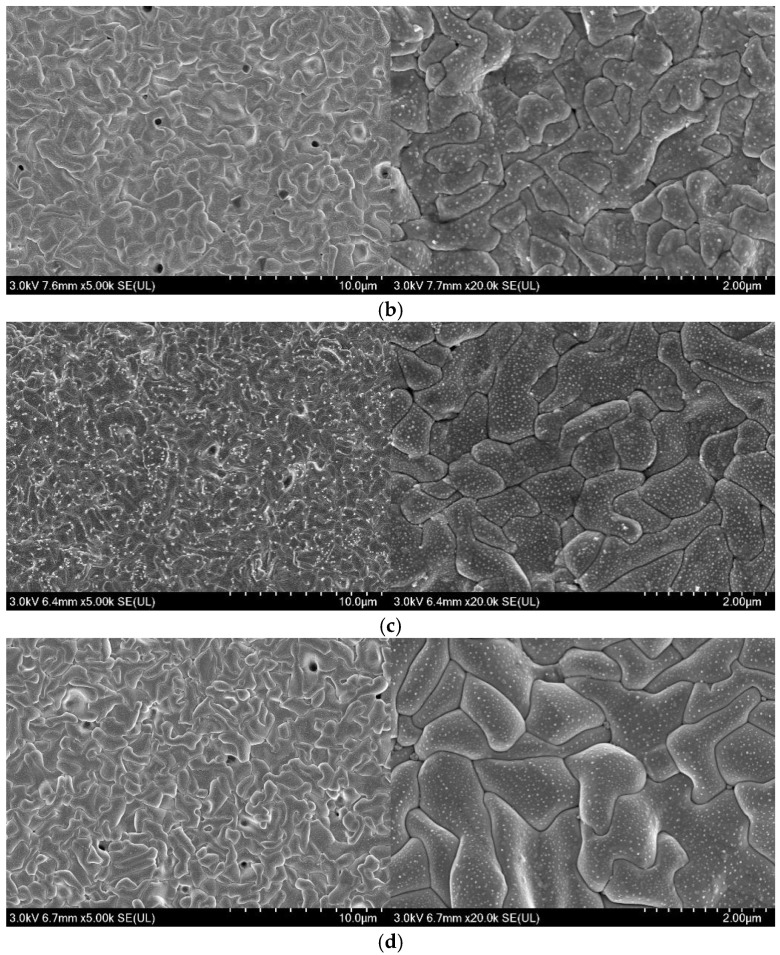
Morphologies of the AgCl film on Ag/AgCl electrodes, (**a**) C-0.1-6, (**b**) C-0.1-12, (**c**) P-0.1-7.5, (**d**) P-0.1-15.

**Figure 8:**
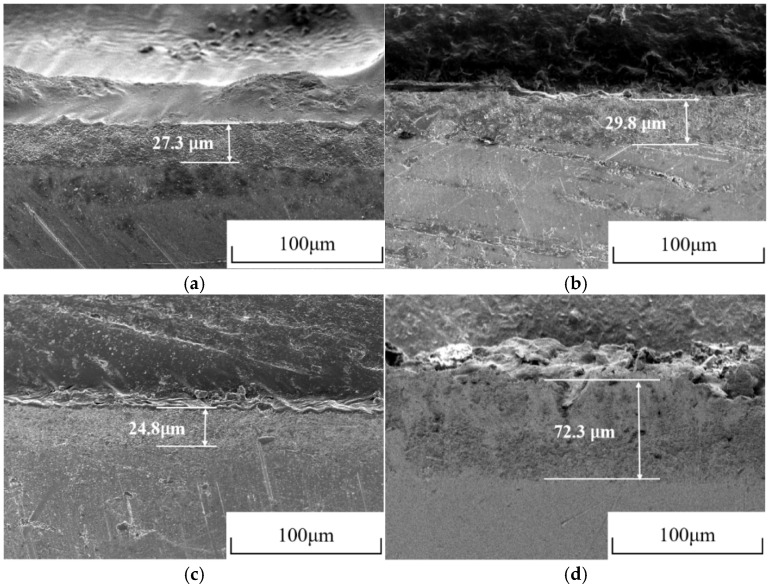
Cross-sections of the AgCl layers on Ag/AgCl electrodes, (**a**) C-0.1-6, (**b**) C-0.1-12, (**c**) C-0.1-7.5, (**d**) P-0.1-15.

Surface morphologies and cross-sections of the Ag/AgCl electrodes were observed by applying SEM, and the results were displayed in Figure 7 and Figure 8, respectively. As Figure 7 shows, the crystal cell of the AgCl film became coarsening, and the density and size of micropores in the surface AgCl film increased, with the increasing electrodeposition time, whether applying the constant current or the pulsed current. In comparison, the density of micropores in the surface AgCl film prepared by applying the pulse current was lower than that in the counterpart prepared by using the constant current under the same equivalent charge transfer conditions, as Figure 7c,d show.

Figure 8 exhibits the cross-section of AgCl films on Ag/AgCl electrodes. Comparative analysis of Figure 8a,b reveals that the thickness of the AgCl film did not significantly increase by extending the electrodeposition time under the constant current electrodeposition conditions. In contrast, the thickness of the AgCl film obviously increased when the deposition duration extended from 7.5 h to 15 h under the pulse current electrodeposition condition. The thickest AgCl film (>70 μm) was observed on the P-0.1-15 Ag/AgCl electrode (Figure 8d), and this thickness was more than twice that of its counterpart C-0.1-12 under the same equivalent charge transfer conditions. The morphology (Figure 7) and cross-section (Figure 8) results confirm that the pulse current electrodeposition can improve the thickness and reduce the density of micropores of the AgCl film, compared to the constant current electrodeposition.

Taking into account the long-term performance (Figure 5 and Figure 6) and the microstructure (Figure 7 and Figure 8), it can be concluded that both the thickness and porosity of the Ag film can affect the lifetime of the Ag/AgCl electrode in concrete environments. In general, the thickness of the Ag film was the more significant factor for the lifetime of Ag/AgCl electrodes, as Figure 7 and Figure 8c,d illustrate.

### 3.5. Composition Analysis of Ag/AgCl Electrodes

**Figure 9:**
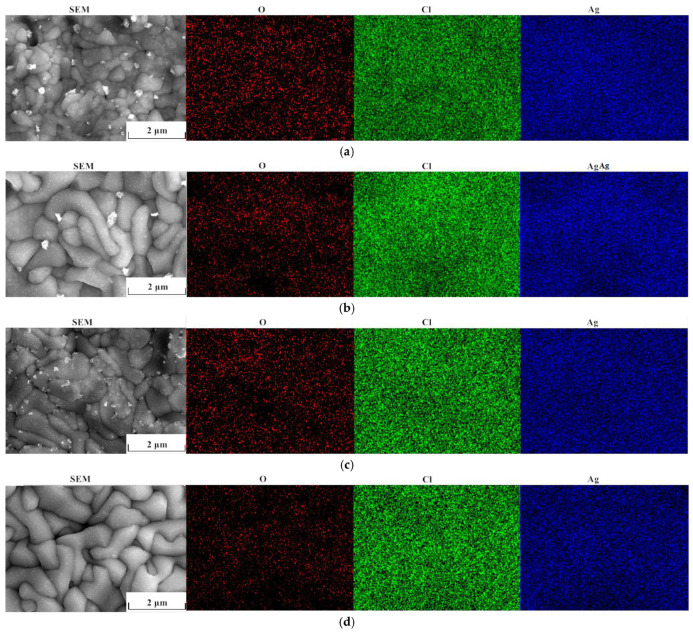
Energy spectrum analysis (EDS) of the surface on Ag/AgCl electrodes, (**a**) C-0.1-6, (**b**) C-0.1-12, (**c**) C-0.1-7.5, (**d**) P-0.1-15.

The contents of O, Cl, and Ag elements of the AgCl film surface on the Ag/AgCl electrode were detected by applying energy dispersive spectroscopy (EDS), and the results are displayed in Figure 9 and Table 4. It is easy to notice that the O, Cl, and Ag are uniformly distributed in the film with the low current density (0.1 mA·cm^−2^) deposition, which is consistent with the results in an earlier study [19]. In comparison, the Cl and Ag on the pulse current electrodeposited sample (Figure 9a,b) are more homogeneous than those on the counterpart electrodeposited with the constant current (Figure 9c,d). In addition, the ratios of Cl/O and Ag/Cl of the AgCl films were analyzed (Table 4). The Cl/O and Ag/Cl ratios were related to the degradation of the AgCl film on the Ag/AgCl electrode. It is easy to notice that the ratios of Cl/O of the AgCl films electrodeposited by applying the pulse current were higher than those of the counterpart films prepared with the constant current under the same equivalent charge transfer conditions. Zhang et al. [12] proposed that the failure of the Ag/AgCl electrode was caused by the OH^−^ penetration through the AgCl film and adsorption on the Ag substrate. Angst et al. [30] ascribed its failure to the AgCl decomposition into Ag_2_O. Wu et al. [31] noticed that the Ag/AgCl electrode with a lower Ag/Cl ratio displayed a longer stable duration in the Cl^−^-containing solution. Thus, a high Cl/O ratio and a low Ag/Cl ratio implies a high stability of the AgCl film on the Ag/AgCl electrode, according to previous studies [12,30,31]. The results displayed in Table 4 suggest that the AgCl films on the Ag/AgCl electrode deposited by using the pulse current were more stable than those of the films prepared by applying the constant current under the same equivalent charge transfer conditions. The stability of the Ag/AgCl electrode prepared by applying the former type of current was enhanced with the extending electrodeposition time.

**Figure 10:**
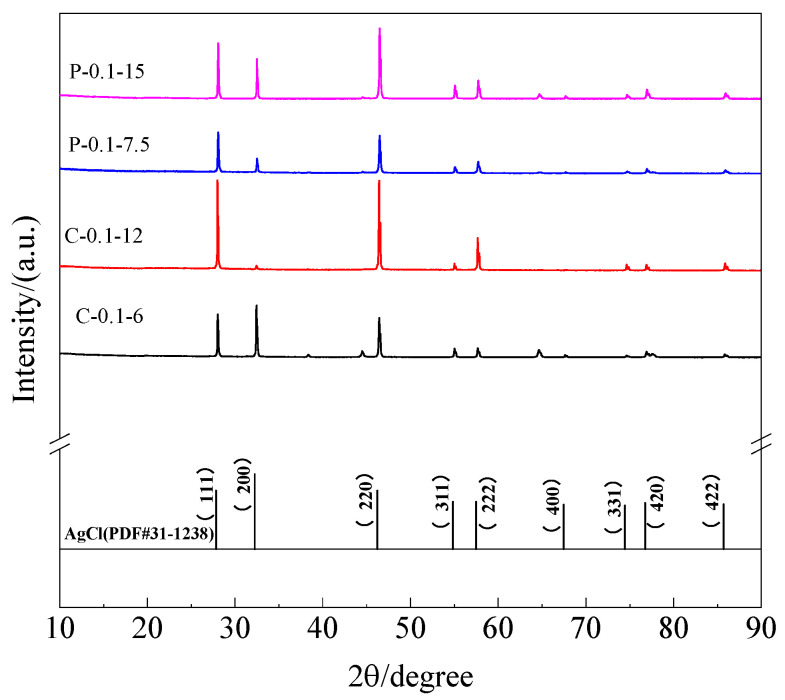
X-ray powder diffractometer (XRD) of the surface on Ag/AgCl electrodes.

The chemical composites of the electrodeposited films also were detected by applying the X-ray diffractometer, and the results are displayed in Figure 10. The electrodeposited products exhibited AgCl crystal structures and no detectable Ag_2_O diffraction peaks were observed, which is consistent with the results reported by Zhang et al. [12]. This situation also may be attributed to the low content of Ag_2_O on the surface of the AgCl film.

### 3.6. Ag/AgCl Electrode Prepared by Pulse Current Deposition and Its Performance

The aforementioned results indicated that the Ag/AgCl electrode prepared by applying the pulse current exhibited better stability (Figure 6 and Figure 7) than its counterpart prepared with the constant current under the same equivalent charge transfer conditions. The performance of the Ag/AgCl electrode was significantly enhanced by the increasing electrodeposition duration with the pulse current, while that of the Ag/AgCl electrode was slightly improved by the extending electrodeposition duration with the constant current (Figure 7). When the Ag/AgCl electrode was prepared by electrodeposition in the 0.1 M HCl solution, a thin layer of AgCl film with some micropores was formed on the electrode surface, as shown in Figure 7. As Figure 7 and Figure 8 show, the C-0.1-12 and P-0.1-7.5 were of similar thickness of AgCl film, while a higher density of micropore formed in the AgCl film on the C-0.1-12 sample. Thus, a higher exchange current density was detected on the C-0.1-12 electrode, as Table 3 shows. The OH^−^ penetrated into the AgCl film and adsorbed on the Ag substrate through the micropores [12], and the AgCl film simultaneously transformed to Ag_2_O and dissolved into the pore solution at the film/solution interface [12,28]. Thus, the Ag/AgCl electrode failed in a short time (Figure 6) due to the rapid degradation of the AgCl film in the pore solution. When the Ag/AgCl electrode was embedded into the mortar, the transformation from AgCl to Ag_2_O was hindered by the low solubility of Ag_2_O in the mortar environment. As a result, the lifetime of the Ag/AgCl electrode in the mortar was significantly longer than the one in the pore solution, as Figure 6 and Figure 7 display.

**Figure 11:**
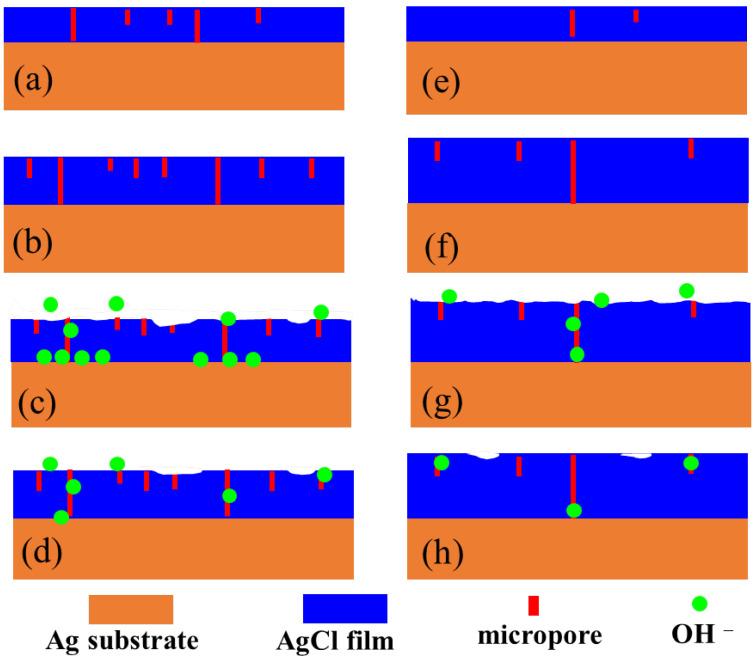
Illustration of the Ag films on the Ag/AgCl electrodes electrodeposited by different currents and their performance in concrete environments, (**a**) constant current electrodeposition over a short time, (**b**) constant current electrodeposition over a long time, (**c**) the long-time constant-current-electrodeposited Ag/AgCl electrode in the simulated pore solution, (**d**) the long-time constant-current-electrodeposited Ag/AgCl electrode in the mortar, (**e**) pulse current electrodeposition over a short time, (**f**) pulse current electrodeposition over a long time, (**g**) the long-time pulse-current-electrodeposited Ag/AgCl electrode in the simulated pore solution, (**h**) the long-time pulse-current-electrodeposited Ag/AgCl electrode in the mortar.

The illustration shown in Figure 11 is adopted to explain the differences between the Ag/AgCl electrodes prepared with pulse currents and the ones electrodeposited by applying constant currents. When the Ag/AgCl electrode was prepared by applying constant current (6 h) and pulse current (7.5 h) over a short time with the same equivalent charge transfers, the AgCl films formed on the Ag substrate with similar thickness and a higher density of micropores can be observed in the former AgCl film (Figure 11a,e). As the electrodeposition duration was doubled, the thickness of the AgCl films prepared by the constant and pulse current were slightly and significantly increasing, respectively. The micropores in the AgCl films also increased, and the density of micropores in the constant current electrodeposited film was higher than that in the pulse-current-electrodeposited counterpart (Figure 11b,f). When Ag/AgCl electrodes were embedded into the pore solution, the OH^−^ adsorbed on the Ag substrate through the micropores [12] and the AgCl film decomposed at the film/solution interface [28]. Therefore, the Ag/AgCl electrode prepared by applying the pulse current, with a thicker AgCl film, displayed a longer lifetime in the pore solution (Figure 11c,g). As the Ag/AgCl electrode was embedded into the mortar, the decomposition of the AgCl film was hindered at the film/mortar interface. Then, the lifetime of the Ag/AgCl electrode was significantly enhanced in comparison to the one in the pore solution, and the pulse-current-electrodeposited Ag/AgCl electrode exhibited a much longer lifetime than the constant-current-prepared sample (Figure 11d,h).

It is worth mentioning that a thicker AgCl film (Figure 8) and a lower density of micropores (Figure 7), as well as a higher Cl/O ratio and lower Ag/Cl ratio (Table 4), are beneficial to the lifetime of the Ag/AgCl electrode in concrete environments. These results, together with Figure 5 and Figure 6, suggest that the thickness of the AgCl film was the determining factor for the lifetime of the Ag/AgCl electrode in concrete.

In general, the Ag/AgCl electrode, which was prepared by applying 15 h of pulse current electrodeposition at 0.1 mA/cm^2^, displayed the best performance, including satisfactory Nernstian response (Figure 3), high exchange current density (Figure 4), and the best long-term stability (Figure 6 and Figure 7). The results also confirm that the pulse current electrodeposition is an effective method for prolonging the lifetime of Ag/AgCl electrodes in concrete environments.

## 4. Conclusions

(1) Under the same equivalent charge transfer conditions, the Ag/AgCl electrodes prepared by applying the pulse current electrodeposition exhibit a wider potential response range and a higher exchange current density than those of the counterpart electrodeposited by the constant current in the identical Cl^−^-containing pore solution.
(2) More micropores can be observed in the electrodeposited AgCl films both by applying the constant current or the pulsed current when the electrodeposition duration was extended. The former AgCl film displayed a higher density of micropores than the latter films under the same equivalent charge transfer conditions. The thickness of the AgCl films were slightly and significantly increased by applying the constant current and the pulsed current when the electrodeposition duration was doubled.
(3) No Ag_2_O can be detected in the AgCl films whether electrodeposition adopted the constant current or the pulsed current. The AgCl films prepared with the former current displayed a lower Cl/O ratio and a higher Ag/Cl ratio than those electrodeposited by applying the latter current.
(4) The lifetimes of the Ag/AgCl electrodes prepared with the pulsed current were longer than those of the counterparts prepared by the constant current, with the same equivalent charge transfers, both in the pore solution and mortar. The lifetime of the Ag/AgCl electrode in the concrete environment was mainly determined by the thickness of the AgCl film.
(5) In general, the Ag/AgCl electrodes prepared using 0.1 mA/cm^2^ pulse current for 15 h displayed the best comprehensive performance, and their lifetimes were about 420 in pore solution and more than 3500 h in mortar. In particular, the P-0.1-15 electrode did not show any significant decrease in OCPs after 3500 h in the mortar without Cl^−^. The lifetime of the Ag/AgCl electrode should be further confirmed in real concrete structures.

## Figures and Tables

**Table 1:** Current parameters applied for the Ag/AgCl electrode in electrodeposition.

Specimen No.	Current Mode	Current Density/ (mA·cm^−2^)	Time/h
C-0.1-6	Constant current Pulse current	0.1	6
C-0.1-12 P-0.1-7.5		0.1 0.1	12 7.5
P-0.1-15		0.1	15

**Table 2:** Fitting results of the Nernst curve for the Ag/AgCl electrodes in the simulated pore solutions with different concentrations of Cl^−^.

Specimen No.	Slope	R^2^
C-0.1-6	−51.131	0.9941
C-0.1-12	−53.846	0.9971
P-0.1-7.5	−56.540	0.9996
P-0.1-15	−54.431	0.9996

**Table 3:** Exchange current density of these Ag/AgCl electrodes calculated from the polarization curves in 0.05 M Cl^−^ simulated pore solutions.

Specimen No.	*i*_0_ (A/cm^2^)
C-0.1-6	3.735 × 10^−5^
C-0.1-12	1.195 × 10^−4^
P-0.1-7.5	4.658 × 10^−5^
P-0.1-15	1.416 × 10^−4^

**Table 4:** Content of elements from EDS on the surface of Ag/AgCl electrodes.

Specimen No.	O/%	Cl/%	Ag/%	Cl/O Ratio	Ag/Cl Ratio
C-0.1-6	3.055	36.588	60.387	11.976	5.042
C-0.1-12	4.375	38.249	57.377	8.7426	6.563
P-0.1-7.5	2.537	31.449	66.014	12.396	5.325
P-0.1-15	2.040	38.517	59.444	18.881	3.148

## Data Availability

The raw data supporting the conclusions of this article will be made available by the authors on request.
